# Expression analysis of genes associated with human osteosarcoma tumors shows correlation of RUNX2 overexpression with poor response to chemotherapy

**DOI:** 10.1186/1471-2407-10-202

**Published:** 2010-05-13

**Authors:** Bekim Sadikovic, Paul Thorner, Susan Chilton-MacNeill, Jeff W Martin, Nilva K Cervigne, Jeremy Squire, Maria Zielenska

**Affiliations:** 1 Department of Pediatric Laboratory Medicine, Pathology Division, Hospital for Sick Children, Toronto, M5G 1X8 Canada; 2 Genetics and Genome Biology Program, Hospital for Sick Children, Toronto, ON, Canada; 3 Division of Applied Molecular Oncology, Ontario Cancer Institute, the University Health Network, Toronto, M5G 2M9 Canada; 4 Department of Pathology and Molecular Medicine, Richardson Labs, Queen's University, Kingston, K7L 3N6 Canada

## Abstract

**Background:**

Human osteosarcoma is the most common pediatric bone tumor. There is limited understanding of the molecular mechanisms underlying osteosarcoma oncogenesis, and a lack of good diagnostic as well as prognostic clinical markers for this disease. Recent discoveries have highlighted a potential role of a number of genes including: *RECQL4*, *DOCK5*, *SPP1*, *RUNX2*, *RB1*, *CDKN1A*, *P53*, *IBSP*, *LSAMP*, *MYC*, *TNFRSF1B*, *BMP2*, *HISTH2BE*, *FOS*, *CCNB1*, and *CDC5L*.

**Methods:**

Our objective was to assess relative expression levels of these 16 genes as potential biomarkers of osteosarcoma oncogenesis and chemotherapy response in human tumors. We performed quantitative expression analysis in a panel of 22 human osteosarcoma tumors with differential response to chemotherapy, and 5 normal human osteoblasts.

**Results:**

*RECQL4*, *SPP1*, *RUNX2*, and *IBSP *were significantly overexpressed, and *DOCK5*, *CDKN1A*, *RB1*, *P53*, and *LSAMP *showed significant loss of expression relative to normal osteoblasts. In addition to being overexpressed in osteosarcoma tumor samples relative to normal osteoblasts, *RUNX2 *was the only gene of the 16 to show significant overexpression in tumors that had a poor response to chemotherapy relative to good responders.

**Conclusion:**

These data underscore the loss of tumor suppressive pathways and activation of specific oncogenic mechanisms associated with osteosarcoma oncogenesis, while drawing attention to the role of *RUNX2 *expression as a potential biomarker of chemotherapy failure in osteosarcoma.

## Background

Osteosarcoma is the most common pediatric tumor of the bone. Clinically, osteosarcoma has a bimodal distribution, with the majority of patients developing the disease during the period of active bone growth in early adolescence. The treatment generally involves surgery, often involving a loss of limb, and adjuvant chemotherapy. The best prognostic marker for osteosarcoma is the response to chemotherapy, where good response to chemotherapy is associated with an overall more favorable patient outcome and survival [1,2].

At the molecular level osteosarcoma is characterized by a high level of genomic instability, highly heterogenous karyotypes both intra- and inter-tumor, and gross changes in gene expression [3-9]. Human osteosarcoma tumors often have osteoblast-like features but may vary within a broad range of epithelial mesenchymal lineages reflective of their poorly differentiated phenotype [10-12]. Therefore, assessment of the molecular changes in osteosarcoma tumors relative to normal osteoblasts can provide important insights concerning gene expression changes associated with both osteosarcoma oncogenesis and with molecular alterations governing differential clinical response to treatment.

The genetic change most commonly associated with osteosarcoma is the loss of the *TP53 *tumor suppressor gene through either genetic mutation or loss of gene expression [13-15]. Patients with Li-Fraumeni syndrome, which results from loss of *TP53*, have a strong predisposition to developing osteosarcoma [16]. Another tumor suppressor gene whose loss of expression is linked to osteosarcoma is *RB1 *[17]. In our recent studies, we have also shown that these genes play a central role in osteosarcoma-related gene expression networks both in human osteosarcoma cell lines [18] and tumor tissues [19]. We used a unique bioinformatic integrative whole-genome approach to map the genetic and epigenetic changes in osteosarcoma tumors and to identify gene networks related to osteosarcoma oncogenesis. Changes that showed the most significant associations with osteosarcoma gene networks included: overexpression and the most significant copy number gain of the chromosome 6p21.1 *RUNX2 *locus, loss of expression and genomic loss of the *DOCK5 *and *TNFRSF10A *loci at chromosome 8p21.1-21.3, and hypomethylation, copy number gain, and overexpression of the *HISTH2BE *gene at chromosome 1q21. Other genes that showed deregulated expression and significant contribution to osteosarcoma gene networks included overexpressed *SPP1*, *IBSP*, *BMP2*, and *c-MYC*, and uderexpressed *CDKN1A*, *LSAMP*, and *CCNB1*. Another gene that is thought to play a role in osteosarcoma and has been shown to be overexpressed is the *FOS *proto-oncogene [20,21]. Similarly, *CDC5L *has been recently proposed as the putative oncogene at the 6p21 locus in osteosarcoma [22]. Finally, a DNA repair gene, *RECQL4*, has been shown to be overexpressed, and its level of overexpression correlates with overall genomic instability in osteosarcoma [23].

Microarray analysis and reverse-transcriptase polymerase chain reaction (RT-PCR) are useful for the molecular classification of tumors and for deriving biological mechanisms that underpin differential prognosis for patients with various types of cancer, including osteosarcoma [24-27]. The use of gene-expression profiling in clinical practice is however limited by the large number of genes that need to be analyzed and by the lack of reproducibility of various array platforms and interpretative methods [28]. Quantitative RT-PCR methods can be readily applied to RNA derived from formalin-fixed, paraffin-embedded (FFPE) pathological specimens, are reproducible and may be highly applicable in clinical practice [29], particularly for a rare tumor such as osteosarcoma in which access to frozen tissue is often limited. RT-PCR can only typically be used to analyze a small number of genes. Therefore it is important to select gene subsets for detailed analyses in which multiple lines of evidence implicate clinical utility. In previous studies, we [18,19], and others [22,30,31] have performed microarray analyses of osteosarcoma cell lines and tissue samples and identified a series of genes with strong potential as biomarkers with clinical utility. Thus, the objective of the current study was to examine expression profiles of *RECQL4*, *DOCK5*, *SPP1*, *RUNX2*, *RB1*, *CDKN1A*, *TP53*, *IBSP*, *LSAMP*, *MYC*, *TNFRSF1B*, *BMP2*, *HISTH2BE*, *FOS*, *CCNB1*, and *CDC5L *genes in a cohort of osteosarcoma tumors and normal human osteoblasts. As a result, we show that *RECQL4*, *SPP1*, *RUNX2*, and *IBSP *are significantly overexpressed, and *DOCK5*, *CDKN1A*, *RB1*, *P53*, and *LSAMP *show significant loss of expression relative to normal osteoblasts. We also show that *RUNX2 *was the only gene with significant overexpression in tumors with an unfavorable response to chemotherapy relative to favorable responders.

## Methods

### Tissue samples

The collection of frozen tissue specimens (n = 15), archival formalin-fixed, paraffin-embedded osteosarcoma sections (n = 7), and clinicopathological data was obtained and handled in accordance with the Hospital for Sick Children Research Ethics guidelines (Toronto, Canada). This was a retrospective study of chemotherapy-naive biopsy samples collected sequentially between 1996 and 2005, and all specimens presented a tumor content higher than 90%. All patients were subjected during treatment to standard regimens for osteosarcoma, comprising cisplatin, doxorubicin, and methotrexate. The patient tumor specimens were revised at the time of study by the pathologist (P.T.). The details of the cases are presented in Table 1. The Huvos grading system was used to rate the level of tumor necrosis following preoperative chemotherapy: Grade I, little or no effect of chemotherapy noted; Grade II, partial response to chemotherapy, with between 50% and 90% necrosis; Grade III, greater than 90% necrosis; and Grade IV, no viable tumor cells are apparent [32]. The good responders are patients with necrosis ≥ 90% [33]. Normal human osteoblasts that were isolated from surgical bone specimens from five healthy individuals were obtained from Promocell (Heidelberg, Germany).

**Table 1:** Descriptive and histopathological features of the tumor cohort and normal osteoblasts.

Sample	Huvos grade	Group	Age	Sex	Site	Histology
HOB A	NA	Normal	NA	M	femur	osteoblasts normal
HOB B	NA	Normal	NA	M	femur	osteoblasts normal
HOB C	NA	Normal	NA	F	femur	osteoblasts normal
HOB D	NA	Normal	NA	M	femur	osteoblasts normal
HOB E	NA	Normal	NA	M	femur	osteoblasts normal

176	III	Good	7	M	humerus	chondroblastic
177	III	Good	10	M	femur	chondroblastic
186	III	Good	7	F	humerus	osteoblastic
255	III	Good	9	M	tibia	osteoblastic
259	III	Good	7	M	femur	osteoblastic
260	III	Good	14	F	femur	osteoblastic
214	IV	Good	4	F	femur	osteoblastic
217	III	Good	10	F	tibia	osteoblastic
220	IV	Good	12	M	tibia	fibroblastic
223	III	Good	12	F	humerus	osteoblastic
230	III	Good	12	F	fibula	osteoblastic

174	II	Poor	14	M	femur	osteoblastic
178	I	Poor	5	F	humerus	osteoblastic
179	I	Poor	11	F	tibia	osteoblastic
182	I	Poor	13	M	femur	osteoblastic
183	II	Poor	12	F	femur	poorly differ.
187	I	Poor	6	F	femur	osteoblastic
234	II	Poor	13	M	femur	osteoblastic
254	I	Poor	7	M	humerus	osteoblastic
256	I	Poor	15	M	femur	poorly differ.
261	I	Poor	13	M	femur	osteoblastic
211	I	Poor	12	F	femur	osteoblastic

### RNA isolation

Total RNA from snap-frozen tissue (5 normal human osteoblasts and 15 tumors) was extracted and purified using the TRIzol Reagent method according to the manufactures protocol (Invitrogen, Carlsbad, CA, USA). FFPE tissues (7 tumors) were deparaffinized with xylene, washed with ETOH, and digested with a proteinase K buffer [34]. Total RNA was extracted and purified as above with TRIzol reagent. The RNA quality was good for all samples as assessed by BioAnalyzer RNA 600 Nano Kit (Agilent Technologies, Palo Alto, CA). Additionally, the overall trends for expression in tumors relative to normal osteoblasts were similar in frozen and FFPE samples corroborating the quality of the extracted RNA (Additional file 1).

### Quantification of mRNA Expression

Quantitative real-time PCR (qRT-PCR) was used to quantify mRNA expression levels of 16 genes (*RUNX2*, *DOCK5*, *TNFRS1B*, *HISTH2BE*, *P21*, *SSP1*, *P53*, *IBSP*, *CCNB1*, *BMP2*, *LSAMP*, *RB1*, *FOS*, *MYC*, *RECQL4*, and *CDC5L*). Briefly, 1-2 ug of total RNA was converted to cDNA using GeneAmp Gold RNA PCR Core Kit (Applied Biosystems, Foster City, CA, USA), as per manufacturers recommendations. Primers were designed to specifically amplify templates of approximately 90-130 nucleotides overlapping exon boundaries of 3' terminal exons using the Primer-Blast software http://www.ncbi.nlm.nih.gov/tools/primer-blast/. The primers were subsequently tested by both PCR and qRT-PCR for specificity and single band amplification. The sequences of the PCR primer pairs used for each gene are shown in Additional file 2. The qRT-PCR assays for a particular gene were undertaken at the same time for all samples under identical conditions, in duplicate. The cycling conditions were as follows: 95°C for 2 min, 40 cycles of 95°C for 15 sec and 60°C for 45 sec, with a final extension 72°C for 5 min.

The mRNA expression levels were determined using Platinum SYBR Green qPCR Supermix-UDG with Rox (Invitrogen, Carlsbad, CA, USA), and the Applied Biosystems Prism 7900 Sequence Detection System (PE Applied Biosystems, Inc., Foster City, CA). The relative expression level of the genes of interest was computed relative to the endogenous control, phosphoglycerate kinase (PGK), to normalize for variances in the quality of RNA and the amount of input cDNA. Additionally, we validated our experimental conditions by analyzing the expression of two genes identified in our previous microarray study [19]; *A2M*, the highest overexpressing gene of the set and our positive control for the current study; and *SLC14A*, the gene with the lowest expression of the set and our negative control for the current study (Additional file 3). The mRNA expression levels for each sample were determined as fold-change values relative to the mean baseline expression levels for five human osteoblasts (HOBs), using the delta delta Ct method of analysis [35].

### Statistical analysis

Results of the delta-delta Ct analysis were log_10 _transformed and imported to Partek Genomic Suite software (Additional file 1). The tumor samples were grouped by Huvos grade into those with favorable response to chemotherapy (Grades I and II) and unfavorable response (Grades III and IV); and were compared either as a group to normal human osteoblasts (i.e. tumor vs. normal), or to each other (i.e. unfavorable vs. favorable). Differences in p-values between groups were obtained using the non-parametric rank-sum Mann-Whitney test, and fold change differences between groups were obtained using the 1-way ANOVA tool (Additional file 4) using the Partek Genomic Suite software. This study was designed as a confirmatory analysis based on specific genes that were previously shown to have significant expression changes in steosarcoma, and thus multiple test correction was not applicable [36]. This analysis is designed to assess the correlation of specific gene expression as an individual parameter against the ostosarcoma phenotype, and is not meant to assess these genes as a group, gene network, or a multiple gene signature.

## Results

The pathology of 22 osteogenic sarcomas and follow-up biopsies were analysed to determine response to chemotherapy and percent of necrotic tissue. This allowed tumors to be grouped as good responders (Huvos grades III and IV with favorable responses to chemotherapy) and poor responders (Huvos grades I and II with unfavorable responses to chemotherapy) (Table 1). Eleven tumors were identified to have 95% or more necrosis as a result of chemotherapy and were labelled as favorable responders, and the remaining 11 tumors were characterized as unfavorable responders. The baseline control consisted of a panel of five normal human osteoblast samples. The majority of tumors displayed osteoblastic histology, and most tumors and all osteoblast controls were of femoral origin.

**Figure 1:**
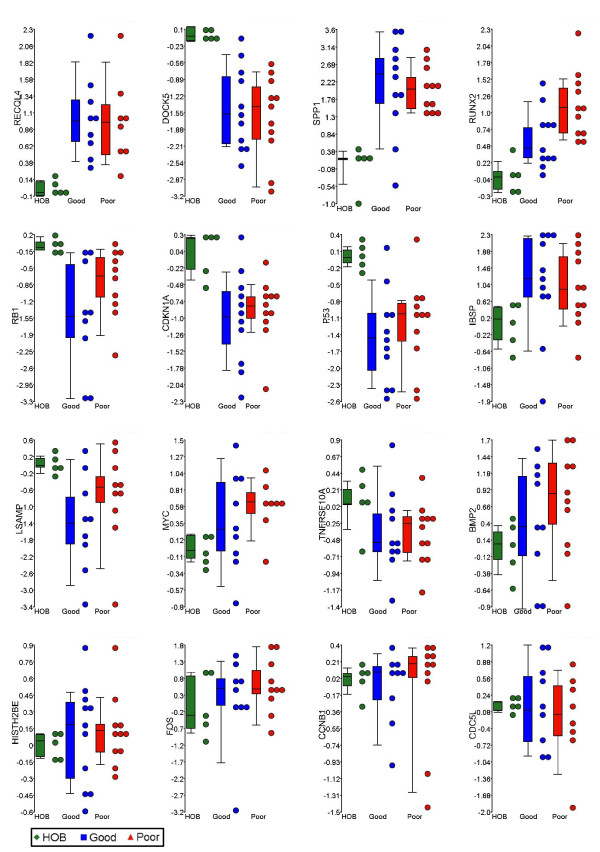
**Expression analysis of osteosarcoma-related genes**. qRT-PCR levels of gene expression of 16 osteosarcoma-related genes in 5 normal human osteoblasts and 22 human osteosarcoma samples are shown on y-axis. The samples are grouped based on the response to chemotherapy status on y-axis. Corresponding box and whiskers plots representing the mean, 25^th ^and 75^th ^percentile (boxes), and 10^th ^and 90^th ^percentile (whiskers) are also shown. From left to right, and top to bottom of the panel, the plots are placed in the order of Mann-Whitney p-value significance (tumor vs. normal). HOB: normal human osteoblasts; Good: Favorable response to chemotherapy (Huvos grades III and IV); Poor: Unfavorable response to chemotherapy (Huvos grades I and II).

In order to quantitatively assess the expression of the target genes (*RECQL4*, *DOCK5*, *SPP1*, *RUNX2*, *RB1*, *CDKN1A*, *TP53*, *IBSP*, *LSAMP*, *MYC*, *TNFRSF1B*, *BMP2*, *HISTH2BE*, *FOS*, *CCNB1*, and *CDC5L*) we performed qRT-PCR on the tumor cohort and human osteoblast samples (Additional file 1). Statistical analysis of these data revealed significant changes in a number of genes (Table 2). Tumors displayed significant overexpression of *RECQL4*, *SPP1*, *RUNX2*, and IBSP genes and loss of expression of *DOCK5*, *CDKN1A*, *RB1*, *TP53*, and *LSAMP *(p < 0.05) (Figure 1). The highest level of overexpression was measured in *SPP1 *with 113-fold overexpression, while the largest reduction of expression of 36-fold was evident in the *DOCK5 *gene. Comparison of tumors with unfavorable response to ch emotherapy to favorable responders revealed *RUNX2 *as the only significant gene (p = 0.03). On average unfavorable responders to chemotherapy showed 3.3-fold increase in the *RUNX2 *gene expression relative to favorable responders. Furthermore, *RUNX2 *expression showed a trend towards overexpression going from normal osteoblasts to favorable responders to chemotherapy and then to unfavorable responders to chemotherapy (Figure 1). The tumor sample #256 that exhibited the worst response to chemotherapy, also showed highest levels (113-fold) of *RUNX2 *overexpression (Additional file 1).

**Table 2:** Statistical evaluation of the tumor-specific and chemotherapy response-related gene expression signatures.

***Gene***	***Tumor vs. Normal***		***Poor vs Good***		***References***
		
	***p-value***	***fold change***	***p-value***	***fold change***	
	
RECQL4	0.00087	10.16	0.84739	-1.14	[22]*
DOCK5	0.00094	-36.81	0.86960	-1.58	[18]
SPP1	0.00180	113.97	0.57674	-1.21	[18]
RUNX2	0.00222	7.13	0.02782	3.30	[18]
RB1	0.00252	-14.87	0.14164	5.14	[16]
CDKN1A	0.00409	-8.61	0.71798	1.27	[18]
P53	0.00409	-20.63	0.45016	1.65	[12-15]
IBSP	0.03382	9.61	0.45016	-1.25	[18]
LSAMP	0.04278	-11.56	0.14164	4.42	[18]
MYC	0.05492	3.05	0.56370	1.61	[17]
TNFRSF10A	0.07359	-2.34	1.00000	-1.07	[18]
BMP2	0.10975	3.86	0.36911	2.21	[18]
HISTH2BE	0.26121	1.30	0.97381	1.10	[18]
FOS	0.33725	2.34	0.56763	2.95	[19,20]
CCNB1	0.41368	-1.20	0.46243	-1.03	[18]
CDC5L	0.90645	-1.13	0.63043	-1.98	[21]

*References are to the original papers describing the change in expression in these genes in osteosarcoma

The remaining genes showed no significant changes in expression in tumors relative to normal osteoblasts. *MYC*, *BMP2*, and *FOS *show an associative trend when overexpressed, and *TNFRSF10A *shows a trend in underexpression in the tumor cohort. Notably, *CDC5L *showed essentially no change in expression levels relative to normal osteoblasts.

## Discussion

Transformation of normal cells and initiation of tumorigenesis involves a combination of genetic and epigenetic changes [37]. Progressive acquisition of such changes ultimately results in destabilization of the genome, deregulation of gene expression pathways and activation of oncogenic gene expression networks. Identification of key genes, or network "nodes", will provide a more comprehensive understanding of tumorigenic processes, and provide more effective diagnostic, prognostic and therapeutic markers. In our recent integrative epi/genomic studies of osteosarcoma cell lines [18] and tumor genomes [19] we identified a number of such genes. A survey of the current literature allowed us to augment this list to a total of sixteen genes whose expression levels were assessed in an expanded tumor cohort. By comparing the gene expression levels to a panel of normal human osteoblasts, it allowed us to identify changes that are likely to be involved in osteosarcoma tumorigenesis. As a result, we identified significant disruptions of gene expression in nine of these genes, including loss of expression of *DOCK5*, *CDKN1A*, *RB1*, *TP53*, and *LSAMP *genes, and overexpression of *RECQL4*, *SPP1*, *RUNX2*, and *IBSP *genes.

Our study demonstrates a significant deregulation of proteins in osteosarcoma that are important effectors in the cell cycle and in differentiation. Significantly, we detected loss of *TP53 *expression, which may also play a role in loss of expression of *CDKN1A*, which encodes the cyclin-dependent kinase inhibitor 1A (p21) and whose expression is activated by p53 [38]. Loss of DOCK5 may play a similar role as it was shown recently that DOCK5 expression is essential for bone differentiation, from precursor osteoclasts [39]. Interestingly, in our recent study we showed that *DOCK5 *is located in the most significant region of copy number loss in osteosarcoma 8p21.2-p21.3 [19] along with the *TNFRSF10A *gene, for which we see an overall trend of loss of expression in our current study. TNFRSF10A is a receptor activated by tumor necrosis factor-related apoptosis inducing ligand TNFSF10 (also known as TRAIL), and is involved in the transduction of cell death signal and induction of cell apoptosis, which is mostly independent of p53 signalling [40].

A significant region of copy number loss at 3q13.31 has been identified in our previous study [19], and has also been observed by another group in 56% of osteosarcomas. This region of copy number loss was shown to correlate with loss of expression and hypermethylation of the *LSAMP *gene, and the authors proposed LSAMP as a novel tumor suppressor in osteosarcoma [31]. Our data agree with these findings and show significant loss of expression of *LSAMP *in the majority of our osteosarcoma samples. *LSAMP *codes for a neuronal surface glycoprotein found in cortical and subcortical regions of the limbic system, but it is currently unclear how this gene may be related to osteosarcoma tumorigenesis.

*RUNX2 *was one of the genes overexpressed in our set of tissue samples, and the only gene whose overexpression was significantly related to poor response to chemotherapy in osteosarcomas. RUNX2 is a member of the Runx transcription factor family consisting of RUNX1, RUNX2, and RUNX3 which function in the development of a number of tissues [41]. Of the three proteins, both RUNX1 and RUNX2 have been associated with oncogenesis. RUNX1 and RUNX2 upregulate LGALS3 (galectin-3) [42], a protein which suppresses anoikis and drug-induced apoptosis [43] and whose expression is correlated with metastasis in osteosarcoma [44] and progression in glioma [45]. Similarly, in lymphoma, overexpression of *RUNX2 *and *MYC *results in the "collaboration" of the two corresponding proteins to attenuate apoptosis and promote proliferation [46]. In developing osteoblasts, expression of *RUNX2 *normally decreases during maturation [47], and overexpression of the gene leads to a higher rate of bone turnover [48]. In bone metastatic breast cancer, RUNX2 promotes cancer cell survival and growth by activating expression of *IHH *and interacts with the TGFβ/BMP signal transduction pathway to parathyroid hormone-related protein (PTHrP) [49]. Thus the finding of elevated expression of *RUNX2 *in osteosarcomas with an unfavorable chemotherapy response is consistent with its oncogenic potential noted in other studies

In addition to *RUNX2*, three other genes, *SPP1*, *RECQL4*, and *IBSP *showed significant overexpression. SPP1 (osteopontin), like RUNX2, is a member of the BMP-signalling protein family. It shows the highest overexpression (113-fold) in our analysis, and has previously been shown to be significantly overexpressed in osteosarcoma tumors [50] and cell lines [51]. Antisense knockdown of *SPP1 *RNA in osteosarcoma cells results in inhibition of *in vivo *tumorigenesis in mice. These findings suggest that overexpression of *SPP1 *plays a role in osteosarcoma tumorigenesis, in particular, in cells lacking expression of cell cycle regulators and differentiation-related genes, as discussed earlier. *RECQL4 *is a gene whose protein product is involved in repair of DNA double stranded breaks and deregulation of its expression was recently shown to be strongly correlated with genomic instability in osteosarcoma [23]. Our data are consistent with this in further reaffirming the association of *RECQL4 *overexpression with osteosarcoma tumorigenesis. The final gene which showed significant overexpression in tumors relative to normal cells, *IBSP*, also known as bone sialoprotein, is a marker of terminal differentiation of bone. In normal osteoblasts RUNX2 and HDAC3 have been shown to suppress IBSP, and upon terminal differentiation loss, of RUNX2 expression derepresses IBSP and allows for terminal differentiation [52]. In tumors, however, although *IBSP *shows significant overexpression relative to osteoblasts, concurrent overexpression of *RUNX2 *indicates possible disruption of the terminal differentiation process.

By comparing our tumor panel, the majority of which (17/22) are the osteoblastic histological subtype, we identified gene expression changes associated with osteosarcoma oncogenesis. However, a possible limitation of this study is that some of the genetic associations described may not be present in osteosarcoma lineages that arise from more primitive cells of origin, including mesenchymal precursors [53-55]. In addition to the associations of gene expression with oncogenesis, we showed that the *RUNX2 *gene displays significant increase in expression in tumors with poor response to chemotherapy relative to the good responders. Our results may be reflective of either increased levels of gene expression in individual tumor cells during disease progression, or alternatively an increased proportion of cell lineages with *RUNX2 *expression in these genetically highly heterogenous cells. Both scenarios would allow selective advantage to the evolving cell lineages during tumorigenesis. Detailed immunohistochemical and imaging experiments will be required to further delineate these possibilities. It also remains to be determined if this correlation is evident at the protein level, and if so, RUNX2 may be a good histological marker for chemotherapy response, which is currently the best predictor of overall outcome in patients with osteosarcoma.

## Conclusion

These data underscore the loss of tumor suppressive pathways the deregulation of cell cycle control proteins, and the activation of specific oncogenic mechanisms associated with osteosarcoma oncogenesis. Our results also draw attention to the role of *RUNX2 *expression as a potential biomarker of chemotherapy failure in osteosarcoma.

## Competing interests

The authors declare that they have no competing interests.

## Authors' contributions

BS: Conceived and designed experiments, performed experiments, analysed data, drafted the manuscript. PT: Performed experiments. SCM: Performed experiments. JM: Revised the manuscript. NKC: Performed experiments, analysed data. JS: Conceived and designed experiments, revised the manuscript. MZ: Conceived and designed experiments, revised the manuscript. All authors have read and approved the final manuscript.

## Pre-publication history

The pre-publication history for this paper can be accessed here:

http://www.biomedcentral.com/1471-2407/10/202/prepub

## Supplementary Material

Additional file 1 **Primer sequences**. Primer sequences for Real-Time PCR Click here for file

Additional file 2 **Gene expression levels in osteosarcoma tumors and normal osteoblasts**. Values of gene expression are represented as delta-delta Ct (log10). Click here for file

Additional file 3 **Controls for qRT-PCR analyses**. Respectively, *A2M *and *SLC14A *were the highest- and lowest- expressed genes in a previous microarray study performed by our group [19], and they were used to validate our conditions for the qRT-PCR experiments of the current study. Click here for file

Additional file 4 **Detailed results of the statistical analyses of gene expression**. Tables represent values calculated using Mann-Whitney and 1-way ANOVA statistical analyses respectively Click here for file
