# Supplementary figures and images for: Expression analysis of genes associated with human osteosarcoma tumors shows correlation of RUNX2 overexpression with poor response to chemotherapy
Source: BMC Cancer. 2010 May 13;10:202. doi: 10.1186/1471-2407-10-202 (PMC2875220; doi:10.1186/1471-2407-10-202)

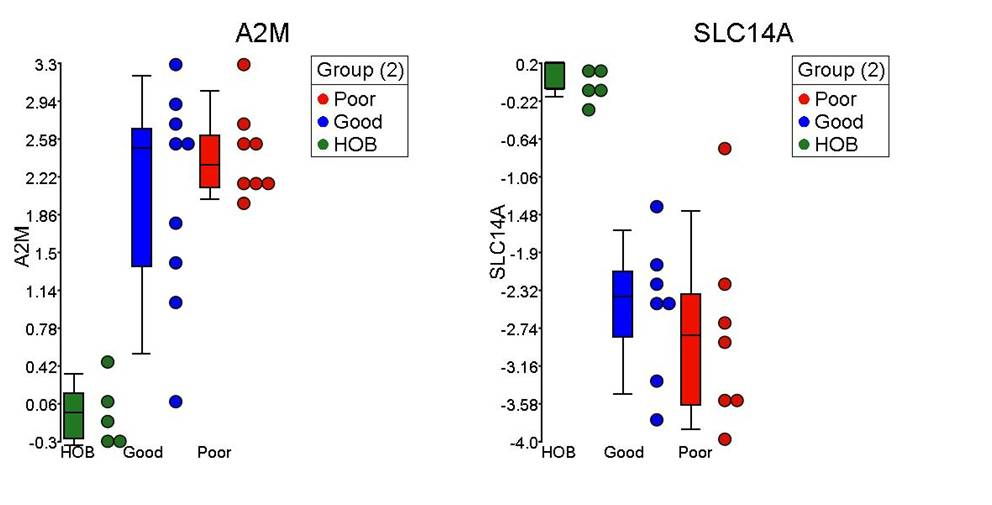

Supplement: Additional file 3 — Controls for qRT-PCR analyses. Respectively, A2M and SLC14A were the highest- and lowest- expressed genes in a previous microarray study performed by our group [19], and they were used to validate our conditions for the qRT-PCR experiments of the current study. [file 1471-2407-10-202-S3.TIFF]
